# PbI_2_‐DMSO Assisted In Situ Growth of Perovskite Wafers for Sensitive Direct X‐Ray Detection

**DOI:** 10.1002/advs.202204512

**Published:** 2022-11-13

**Authors:** Wenjun Liu, Tongyu Shi, Jiongtao Zhu, Zhenyu Zhang, Dong Li, Xingchen He, Xiongsheng Fan, Lingqiang Meng, Jiahong Wang, Rui He, Yongshuai Ge, Yanliang Liu, Paul K. Chu, Xue‐Feng Yu

**Affiliations:** ^1^ Materials Interfaces Center Shenzhen Institute of Advanced Technology Chinese Academy of Sciences Shenzhen Guangdong 518055 China; ^2^ Nano Science and Technology Institute University of Science and Technology of China Suzhou 215123 China; ^3^ University of Chinese Academy of Sciences Beijing 100049 P. R. China; ^4^ Research Center for Medical Artificial Intelligence Shenzhen Institute of Advanced Technology Chinese Academy of Sciences Shenzhen 518055 China; ^5^ Department of Physics Department of Materials Science and Engineering and Department of Biomedical Engineering City University of Hong Kong Tat Chee Avenue, Kowloon Hong Kong China

**Keywords:** defects density, in situ growth, PbI_2_‐DMSO, perovskite wafer, perovskite X‐ray detection

## Abstract

Although perovskite wafers with a scalable size and thickness are suitable for direct X‐ray detection, polycrystalline perovskite wafers have drawbacks such as the high defect density, defective grain boundaries, and low crystallinity. Herein, PbI_2_‐DMSO powders are introduced into the MAPbI_3_ wafer to facilitate crystal growth. The PbI_2_ powders absorb a certain amount of DMSO to form the PbI_2_‐DMSO powders and PbI_2_‐DMSO is converted back into PbI_2_ under heating while releasing DMSO vapor. During isostatic pressing of the MAPbI_3_ wafer with the PbI_2_‐DMSO solid additive, the released DMSO vapor facilitates in situ growth in the MAPbI_3_ wafer with enhanced crystallinity and reduced defect density. A dense and compact MAPbI_3_ wafer with a high mobility‐lifetime (µ*τ*) product of 8.70 × 10^−4^ cm^2^ V^−1^ is produced. The MAPbI_3_‐based direct X‐ray detector fabricated for demonstration shows a high sensitivity of 1.58 × 10^4^ µC Gyair^−1^ cm^−2^ and a low detection limit of 410 nGy_air_ s^−1^.

## Graphical abstract

PbI_2_‐DMSO powders are adopted as a solid additive for isostatic pressing of MAPbI_3_ wafers to promote in situ crystal growth. A dense and compact MAPbI_3_ wafer exhibits high mobility‐lifetime product of 8.70 × 10^−4^ cm^2^ V^–1^. The X‐ray detector shows high sensitivity of 1.58 × 10^4^ µC Gy_air_^–1^ cm^–2^ and low detection limit of 410 nGy_air_ s^–1^.

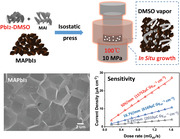

## 1 Introduction

X‐ray detection and imaging have many applications such as medical imaging, industrial detection, safety inspection, and materials testing.^[^ ^1^ ^]^ Recently, metal halide perovskite materials with desirable properties such as heavy element composition, high X‐ray absorption, low defect density, and large mobility‐lifetime (µ*τ*) product^[^ ^2^ ^]^ are emerging as advanced candidates for high‐sensitivity direct X‐ray detection.^[^ ^3^ ^]^ In 2013, Kanatzidis M.G et al. demonstrated CsPbBr_3_ perovskite single crystals for direct X‐ray detection with resolution comparable to that of commercial CdZnTe.^[^ ^4^ ^]^ Huang and Wei et al. reported a sensitive single‐crystal MAPbBr_3_ X‐ray detector with high sensitivity and low detection limits,^[^ ^2^, ^5^ ^]^ and Liu et al. produced a lead‐free perovskite single‐crystal X‐ray detector.^[^ ^6^ ^]^ However, the growth of large perovskite single crystals is complex and time‐consuming thus rendering direct large‐area X‐ray imaging challenging.^[^ ^7^ ^]^ In general, large‐area perovskite thin films can be fabricated by solution‐based processes developed for solar cells and light‐emitting diodes^[^ ^8^ ^]^ but owing to the low solubility of perovskite precursors, solution processes may not be suitable for thick (hundreds of micrometer) perovskite films intended for high‐energy X‐ray absorption.^[^ ^9^ ^]^

In comparison, polycrystalline perovskite wafers prepared by simple isostatic pressing have scalable size and thickness^[^ ^3^, ^10^ ^]^ boding well for direct X‐ray detection and imaging.^[^ ^10b^ ^]^ In 2017, Brabec et al. proposed an MAPbI_3_‐based X‐ray detector with a high sensitivity of 2527 µC Gy_air_^−1^cm^−2^,^[^ ^11^ ^]^ and Tang et al. fabricated a large‐area, pinhole‐free Cs_2_AgBiBr_6_ wafer using the static pressure method as well.^[^ ^12^ ^]^ Wei et al. have synthesized large‐area 2D wafers by rapid tableting with excellent X‐ray detection properties as well as operational stability comparable to those of 2D perovskite single crystals.^[^ ^13^ ^]^ However, the isostatic‐pressed perovskite wafer always surfaces from internal vacancy and lots of grain boundaries due to the insufficient perovskite crystal growth in the solid phase reaction, and high temperature or pressure is usually required to produce dense and wafer‐scale perovskite materials for efficient X‐ray absorption and charge transport.^[^ ^14^ ^]^ Unfortunately, the high temperature inevitably damages the perovskite crystals giving rise to a large charge trap density^[^ ^15^ ^]^ consequently impeding integration of perovskite wafers into flat X‐ray imaging panels.^[^ ^16^ ^]^ Although the solution (DMSO/DMF) strategy has been adopted to promote the growth of crystalline perovskite thin films,^[^ ^17^ ^]^ in the solid state preparation of thick perovskite wafer, the DMSO solvent additive is not able to directly add to the perovskite powders, and the DMSO vapor treatment is also seldom used to prepare perovskite wafers because the vapor generated cannot penetrate thick perovskite wafers. Therefore, it is imperative to design a moderate solution method to foster the growth of thick crystalline perovskite wafers intended for X‐ray detection.

In this study, PbI_2_‐DMSO powders are adopted as a solid additive for isostatic pressing of MAPbI_3_ perovskite wafers to promote crystal growth. The white PbI_2_‐DMSO powders that can be synthesized by complexing PbI_2_ and DMSO are converted into PbI_2_ during annealing while releasing DMSO vapor. During isostatic pressing, PbI_2_‐DMSO, MAI, and MAPbI_3_ are mixed and squashed to the perovskite wafer. At a low annealing temperature, the DMSO vapor released from PbI_2_‐DMSO leads to in situ crystal growth in the interior of the perovskite wafer resulting in enlarged grain sizes, passivated grain‐boundaries, and reduced defect densities. The dense and compact MAPbI_3_ wafer is demonstrated to have excellent optoelectronic properties such as a large µ*τ* product of 8.70 × 10^−4^ cm^2^V^−1^. The PbI_2_‐DMSO assisted MAPbI_3_ wafer shows a high sensitivity of 1.58×10^4^ µC Gy_air_^−1^ cm^−2^and a low detection limit of 410 nGy_air_ s^−1^ in X‐ray detection, which is comparable to those of perovskite single‐crystal based devices.

## 2 Results and Discussion

**Figure 1:**
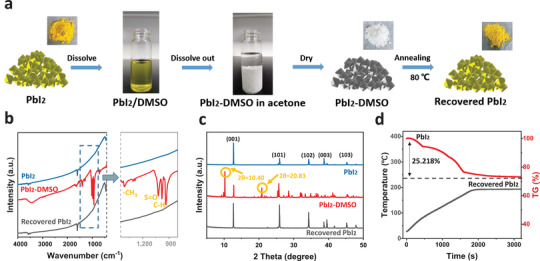
a) Schematic illustration of the preparation of the PbI_2_‐DMSO powder; b) FTIR and c) XRD spectra of PbI_2_, PbI_2_‐DMSO and annealed PbI_2_‐DMSO; d) TG spectra of the PbI_2_‐DMSO powder.

DMSO is a common solvent or additive in preparing perovskite photoelectric devices to promote the growth of perovskite crystals.^[^ ^18^ ^]^ Herein, PbI_2_‐DMSO powders serve as the solid additive to facilitate in situ growth of the MAPbI_3_ wafer during isostatic pressing. Preparation of the PbI_2_‐DMSO powder is illustrated in **Figure** **1**a and Figure S1, Supporting Information. The yellow PbI_2_ powders are dissolved in DMSO and extracted from acetone and after drying, white PbI_2_‐DMSO powders are obtained. The white PbI_2_‐DMSO powders are reconverted into yellow PbI_2_ powders by annealing at 80 °C and DMSO vapor is emitted. Figure 1b presents the FTIR spectra of the pristine PbI_2_, PbI_2_‐DMSO, and recovered PbI_2._ PbI_2_‐DMSO shows peaks at 935, 983, and 1307 cm^−1^ consistent with C–H bonding in DMSO^[^ ^17^, ^19^ ^]^ and the PbI_2_‐DMSO powder exhibits peaks at 1025 and 150 cm^−1^ associated with the S=0 cage.^[^ ^17b^ ^]^ In contrast to pure DMSO, the S=O peak from PbI_2_‐DMSO shifts from 1053 to 1025 cm^−1^ indicating coordination of DMSO with PbI_2_, and formation of PbI_2_‐DMSO powders.^[^ ^20^ ^]^

The PbI_2_‐DMSO powders exhibit X‐ray diffraction (XRD) spectra different from those of the original PbI_2_ and recovered PbI_2_ as shown in Figure 1c. The peaks from PbI_2_ and recovered PbI_2_ are in agreement with those from PbI_2_ at 2*θ* = 12.77◦ (001), 25.91◦ (101), 34.32◦ (102), 38.72◦ (003), 39.73◦ (110), 41.67◦ (111), 45.04◦ (103), 48.01◦ (201).^[^ ^21^ ^]^ The recovered PbI_2_ exhibits higher XRD peak intensity than the original PbI_2_ indicative of enhanced crystallinity. The additional peaks (2*θ* = 10.40◦, 20.83◦) from PbI_2_‐DMSO stem from PbI_2_‐(DMSO)_2_,^[^ ^2^, ^17^ ^]^ and the recovered PbI_2_ show the same Raman spectra as the original PbI_2_ powder as shown in Figure S2, Supporting Information.^[^ ^22^ ^]^ Figure S3, Supporting Information shows the morphology of the original PbI_2_, PbI_2_‐DMSO, and recovered PbI_2_ powders obtained by scanning electron microscopy (SEM). The PbI_2_ and PbI_2_‐DMSO complex exhibit different UV‐visible spectra as shown in Figure S4, Supporting Information. The thermogravimetric analysis (TG) of the PbI_2_‐DMSO complex powder in Figure 1d reveals thermal stability below 80 °C, but it starts to lose weight at higher a temperature and finally converted into a yellow PbI_2_ powder. The released DMSO vapor is expected to promote crystal growth of the perovskite materials.^[^ ^23^ ^]^ PbI_2_‐DMSO loses about 25.22% of its weight to form recovered PbI_2_. The composition of this PbI_2_‐DMSO complex powder is calculated to be PbI_2_‐(DMSO)_2_.^[^ ^17b^ ^]^

**Figure 2:**
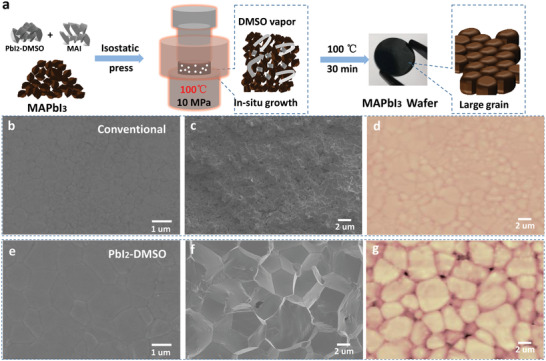
a) Schematic illustration of PbI_2_‐DMSO assisted isostatic pressing of the MAPbI_3_ wafer; b,e) SEM images, c,f) Cross‐sectional SEM images, and d,g) AFM images of the conventional and PbI_2_‐DMSO assisted MAPbI_3_ wafers.

The PbI_2_‐DMSO powders serve as a solid additive during isostatic pressing of the MAPbI_3_ perovskite wafers as shown in **Figure** **2**a. The MAPbI_3_ perovskite powder with a spot of PbI_2_‐DMSO (1% weight ratio) and an equal amount of MAI is squashed onto the perovskite wafer at 100 °C and 10 MPa for 60 min. During hot isostatic pressing, PbI_2_‐DMSO decomposes into PbI_2_ and DMSO vapor and PbI_2_ reacts with MAI to form MAPbI_3_. The DMSO vapor released from the MAPbI_3_ wafer is expected to promote in situ growth of perovskite crystals with larger grain size and enhanced crystallinity. Subsequently, the MAPbI_3_ wafer is heated on hotplate to 100 °C for 30 min to evaporate the DMSO vapor forming the MAPbI_3_ full wafer.

The morphology of the conventional MAPbI_3_ wafer without the PbI_2_‐DMSO additive and PbI_2_‐DMSO assisted MAPbI_3_ wafer is examined by SEM and AFM as shown in Figure 2b–g. The conventional MAPbI_3_ wafer contains small crystals with a lot of grain boundaries and the interior of the conventional MAPbI_3_ wafer contains many grain boundaries and voids that inhibit X‐ray absorption and charge transport and collection in the MAPbI_3_ wafer. In contrast, the PbI_2_‐DMSO‐assisted MAPbI_3_ wafer has a larger grain size and less grain boundaries and the interior also shows a dense and compact morphology without voids. This is because the DMSO vapor facilitates in situ growth of the MAPbI_3_ perovskite crystals. For investigating the behind mechanism of the PbI_2_‐DMSO induced in situ crystal growth of MAPbI_3_ wafer, the SEM images and XRD pattern of the intermediate phase of the PbI_2_‐DMSO assisted MAPbI_3_ wafer before annealing were shown in Figure S5, Supporting Information, and the intermediate MAPbI_3_ wafer exhibits rod‐shaped MAI‐PbI_2_‐DMSO intermediate phase at the grain boundaries with tiny X‐ray diffraction peaks. Under annealing, the MAI‐PbI_2_‐DMSO intermediate phase transforms into MAPbI_3_, releasing DMSO vapor, and the adjacent small grains are merged, resulting in enlarged grain size and enhanced crystallinity of MAPbI_3_ materials, which is consistent with previous reports.^[^ ^17^, ^24^ ^]^ The PbI_2_‐DMSO assisted MAPbI_3_ wafer exhibits lower defect trap density of 1.22 × 10^10^ cm^−3^ than the conventional sample with defect trap density of 1.63 × 10^10^ cm^−3^, and it also has higher carrier mobility of 3.5 cm^2^ V^−1^ S^−1^ than the conventional device (1.3 cm^2^ V^−1^ S^−1^) according to the space charge‐limited current measurement as shown in Figure S6, Supporting Information. Both the conventional and PbI_2_‐DMSO‐assisted MAPbI_3_ wafers show a uniform surface morphology with comparable roughness of 37.90 and 39.20 nm as shown in Figure S7, Supporting Information, respectively. However, excessive PbI_2_‐DMSO additive can result in a rough and incomplete surface morphology on the MAPbI_3_ wafer as shown in Figure S8, Supporting Information, and the PbI_2_‐DMSO assisted MAPbI_3_ wafers with 10% additive exhibit an uneven surface with roughness up to 206.7 nm as shown in Figure S9, Supporting Information. The wafer roughness parameters are listed in Table S1, Supporting Information. The results confirm that the PbI_2_‐DMSO additive facilitates in situ crystal growth of the MAPbI_3_ wafer with better optical, electrical, and X‐ray detection properties. In addition, as shown in Figure S10, Supporting Information, the PbBr_2_‐DMSO powders were also successfully synthesized by using same method with PbI_2_‐DMSO, and the PbBr_2_‐DMSO powders serve as a solid additive during isostatic pressing of the MAPbBr_3_ perovskite wafers, and it is able to significantly enlarge the MAPbBr_3_ grain size and enhance the crystallinity of MAPbBr_3_ perovskite wafers. That indicates this strategy is also able to be applied in other perovskite wafers fabrication, and this make it more general to prepare highly crystalline perovskite wafers.

**Figure 3:**
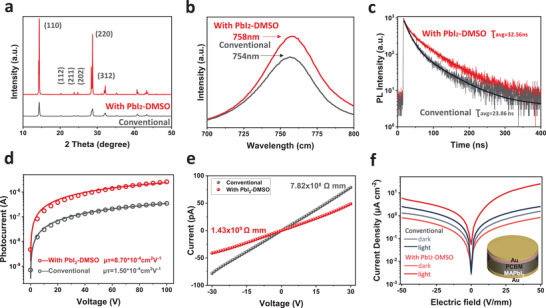
a) XRD patterns, b) PL spectra, c) TRPL, d) Photoconductivity, and (e) Resistivity of the conventional and PbI_2_‐DMSO assisted MAPbI_3_ wafers; f) *J*–*V* characteristics of the conventional and PbI_2_‐DMSO assisted MAPbI_3_ detectors.

**Figure** **3**a presents the XRD spectra of the conventional and PbI_2_‐DMSO assisted MAPbI_3_ perovskite wafers disclosing two main peaks assigned to the (110) and (220) planes of MAPbI_3_.^[^ ^25^ ^]^ The PbI_2_‐DMSO‐assisted MAPbI_3_ wafer shows higher XRD intensity for the (110) and (220) peaks than the conventional wafer suggesting better crystallinity in line with the SEM images. The steady‐state photoluminescence (PL) and time‐resolved photoluminescence (TRPL) properties of the conventional and PbI_2_‐DMSO assisted MAPbI_3_ perovskite wafers are examined to determine the optical properties as shown in Figure 3b,c. The PbI_2_‐DMSO assisted MAPbI_3_ wafer exhibits significantly higher PL intensity than the conventional MAPbI_3_ wafer and the PL peak red‐shifts from 754 to 758 nm confirming that reduced defect density and enhanced crystallinity. The TRPL of the conventional and PbI_2_‐DMSO assisted MAPbI_3_ perovskite wafer can be fitted with an exponential decay function, where the A_1_ and A_2_ are the fractional contributions of the PL decay lifetime. A short lifetime (*τ*_1_) corresponds to fast decay related to quenching (including charge transfer) and defects and a long lifetime (*τ*_2_) corresponds to slower decay related to recombination. The conventional MAPbI_3_ wafer has a short lifetime *τ*_1_ = 24.31 ns and long lifetime *τ*_2_ = 55.40 ns but in comparison, the PbI_2_‐DMSO assisted MAPbI_3_ wafer shows a longer short lifetime *τ*_1_ = 54.75 ns and long lifetime *τ*_2_ = 68.60 ns. Furthermore, *τ*_ave_ improves from 23.86 to 32.56 ns as a result of the reduced defect density and enhanced carrier transport in the MAPbI_3_ wafer. The TRPL parameters are listed in Table S2, Supporting Information. Hence, the PbI_2_‐DMSO‐assisted MAPbI_3_ wafer has significantly better crystallinity and smaller defect density than the conventional MAPbI_3_ wafer, which are expected to benefit charge transport and collection in the MAPbI_3_ wafer.

The overall charge collection characteristics of the conventional and PbI_2_‐DMSO assisted MAPbI_3_ wafers are studied byassessing the photoconductivity as shown in Figure 3d and the µ*τ* product can be obtained by fitting the voltage‐dependent photocurrents according to the following Hecht equation^[^ ^26^ ^]^:

(1) $$I=I_{0}\mu \tau V/L^{2}\left(1-\exp\left(-L^{2}/\mu \tau V\right)\right),$$

where *I*_0_ is the saturated current, L is the MAPbI_3_ wafer thickness, and V is the applied voltage. The µ*τ* products of the conventional and PbI_2_‐DMSO assisted MAPbI_3_ wafers are determined to be 1.51 × 10^−4^ cm^2^ V^−1^ and 8.70 × 10^−4^ cm^2^ V^−1^, respectively. The enhanced µ*τ* products of PbI_2_‐DMSO assisted MAPbI_3_ wafers stem from the DMSO vapor that facilities in situ growth of MAPbI_3_ with high crystallinity, large grain size, and low defect density. The resistivity of the conventional and PbI_2_‐DMSO‐assisted MAPbI_3_ wafers are derived and shown in Figure 3e. The PbI_2_‐DMSO‐assisted MAPbI_3_ wafer has a high resistivity of 1.43 × 10^9^ Ω cm, which is two times larger than that of the conventional wafer of 7.82 × 10^8^ Ω cm. The resistivity enhancement is attributed to suppressed ionic migration and defects and is beneficial to reducing the dark current and noise during X‐ray detection. Figure 3f shows the current density‐voltage (*J‐*‐*V*) characteristics of the conventional and PbI_2_‐DMSO assisted MAPbI_3_ wafers based on the direct conversion X‐ray detectors with a configuration of Au/MAPbI_3_/PCBM/Au and good energy level alignment (Figure S11, Supporting Information). The MAPbI_3_ wafer is about 600 µm thick as shown in Figure S12, Supporting Information. The PbI_2_‐DMSO assisted detector shows obviously smaller dark current and bigger light current than the conventional detector corroborating better X‐ray detection. The dark current density of the PbI_2_‐DMSO assisted detector is only 1.51 µA cm^−2^ in contrast to the dark current of 2.52 µA cm^−2^ in the conventional detector at electric field of 50 V mm^−1^. It is because of suppressed ionic migration and reduced defect density in the PbI_2_‐DMSO‐assisted MAPbI_3_ wafer. The switching ratios of the devices are presented in Figure S13, Supporting Information, and the PbI_2_‐DMSO‐assisted perovskite X‐ray detector delivers better detection performance.

**Figure 4:**
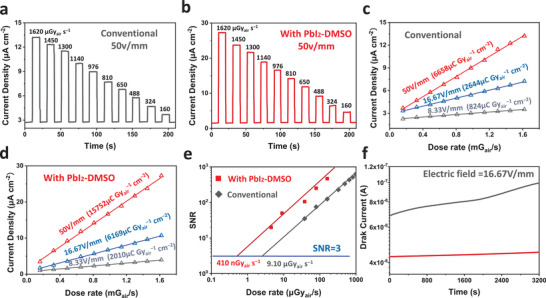
X‐ray response profiles of a) Conventional and b) PbI_2_‐DMSO‐assisted MAPbI_3_ X‐ray detectors; X‐ray current densities as a function of dose rate at different applied electric fields: c) Conventional detector and d) PbI_2_‐DMSO‐assisted detector; e) dose rate dependent SNR of the detectors; f) Temporal baseline tracking of the detectors.

To obtain more information about X‐ray detection, the time‐resolved current densities of the conventional and PbI_2_‐DMSO assisted MAPbI_3_ X‐ray detectors for various X‐ray dose rates from 1620 µGy_air_ s^−1^ to 160 µGy_air_ s^−1^ are determined and presented in **Figure** **4**a,b. The photocurrent densities show a good linear relationship with the X‐ray dose rates. The PbI_2_‐DMSO‐assisted detector shows smaller dark current densities and larger X‐ray on‐off ratios than the conventional detectors. Figure S14, Supporting Information displays the current response upon X‐ray on/off for the conventional and PbI_2_‐DMSO assisted detectors at different electric fields. In order to quantify the sensitivity of the detectors, the X‐ray‐generated current densities are shown as a function of the dose rates in Figure 4c,d) and the sensitivity is derived from the slope of the photocurrent densities versus X‐ray dose rates. The conventional detector shows X‐ray sensitivities of 0.82 × 10^3^, 2.64 × 10^3^, and 6.66 × 10^3^ µCGy_air_^−1^ cm^−2^ at electric fields of 8.33, 16.67, and 50 V mm^−1^, respectively. In contrast, the PbI_2_‐DMSO assisted detector shows appreciably better sensitivities of 2.01 × 10^3^, 6.17 × 10^3^, and 1.58 × 10^4^ µCGy_air_^−1^ cm^−2^ at electric fields of 8.33, 16.67, and 50 V mm^−1^, respectively.

To further investigate the detector properties, the signal‐to‐noise ratios (SNR) of both detectors are determined by the following equation:

(2) $$\mathrm{SNR}=I_{\mathrm{signal}}/I_{\mathrm{noise}}=\left(I_{\mathrm{photo}}-I_{\mathrm{dark}}\right)/I_{\mathrm{noise}}$$

where *I*_signal_ is the signal, *I*_noise_ is the noise, *I*_photo_ is the average current under X‐ray irradiation, and *I*_dark_ is the average dark current calculated from several parallel experiments at each bias. The SNR values are plotted as a function of dose rates in Figure 4e. A large electric field of 50 V mm^−1^ is applied to the opposite electrodes when extracting the X‐ray‐generated charges from the interior of the thick MAPbI_3_ wafer. The SNR values exhibit a linear dependence with X‐ray dose rates and the suitable should maintain an SNR of over three according to the International Union of Pure and Applied Chemistry standard. As expected, the PbI_2_‐DMSO assisted detector shows a lower limit‐of‐detection (LoD) of 410 nGy_air_ s^−1^ compared to the conventional detector with a LoD of 9.10 µGy_air_ s^−1^. The current drift (*I*_drift_) is another vital parameter determined by the following equation:

(3) $$I_{\mathrm{drift}}=\left(I_{t}-I_{0}\right)/\left(E\times S\times t\right),$$

where *I*_t_ is the current at time *t*, *I*_0_ is the current immediately after stabilization, *E* is the electric field, and *S* is the device area. As shown in Figure 4f, *I*_drift_ of the PbI_2_‐DMSO assisted detector is 1.31 × 10^−4^ nA cm^−1^ s^−1^ V^−1^, which is clearly less than that of the conventional detector (1.85 × 10^−3^ nA cm^−1^ s^−1^ V^−1^). The improvement stems from suppressed ion migration in the PbI_2_‐DMSO‐assisted MAPbI_3_ wafer with a large grain size and defect density. The ion migration of perovskite wafer was investigated through Kelvin Probe Force Microscopy measurement in Figure S15, Supporting Information, the PbI_2_‐DMSO assisted MAPbI_3_ wafer exhibits significantly lower electric potential than the conventional sample, indicating suppressed ion migration, which is consistant with previous reports.^[^ ^27^ ^]^

**Figure 5:**
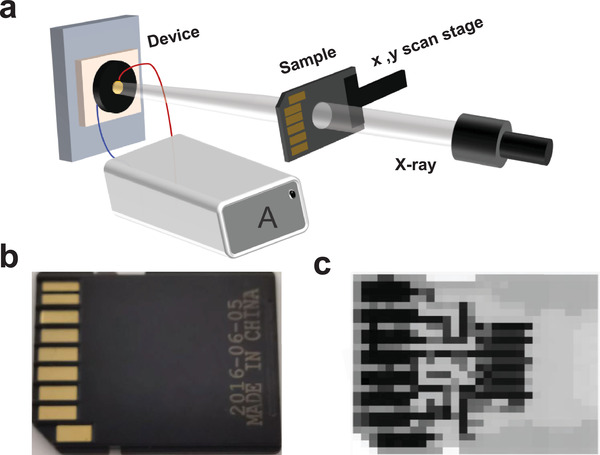
a) Schematic illustration of the setup for X‐ray imaging; b) Photograph and c) X‐ray image of a memory card.

To evaluate the stability of the X‐ray detectors, cycling tests are performed by turning the incident X‐ray on and off. The PbI_2_‐DMSO assisted perovskite X‐ray detector exhibits excellent stability with very little change in the signal after 1 h exposure at a large dose rate of 1.62 mGy_air_ s^−1^ as shown in Figure S16, Supporting Information. For further improving the stability of the perovskite X‐ray detector, the stability of perovskite materials under ambient conditions, high energy X‐ray radiation, and high electrical field should be extensively studied and improved before commercialization. As shown in the demonstration outlined in **Figure** **5**a, a memory card consisting of a plastic case and metal core is tested and the results are presented in Figure 5b which reveals a clear outline of the memory card compared to operating the X‐ray detector in the scanning mode shown in Figure 5c.

## 3 Conclusion

PbI_2_‐DMSO powders are used as the additive to facilitate crystal growth of MAPbI_3_ and can be easily synthesized by complexing PbI_2_ and DMSO. During isostatic pressing of the MAPbI_3_ wafer, the DMSO vapor released from PbI_2_‐DMSO facilitates in situ growth in the interior of the perovskite wafer leading to larger grain sizes, passivated grain boundaries, and reduced defect density. The dense and compact MAPbI_3_ wafer shows a large µ*τ* product of 8.70 × 10^−4^ cm^2^V^−1^ and the MAPbI_3_‐based X‐ray detector has a high sensitivity of 1.58 × 10^4^ µC Gy_air_^−1^ cm^−2^ and low detection limit of 410 nGy_air_ s^−1^. This study demonstrates an efficient strategy to prepare high‐quality perovskite wafers for X‐ray detection with high sensitivity and low detection limit, rendering the device competitive in commercial X‐ray detection.

## Conflict of Interest

The authors declare no conflict of interest.

## Supporting information

Supporting Information

## Data Availability

The data that support the findings of this study are available from the corresponding author upon reasonable request.
