# Supplementary material for: PbI2‐DMSO Assisted In Situ Growth of Perovskite Wafers for Sensitive Direct X‐Ray Detection
Source: Adv Sci (Weinh). 2022 Nov 13;10(1):2204512. doi: 10.1002/advs.202204512 (PMC9811467; doi:10.1002/advs.202204512)
Supplement: Supplementary file 1 — Supporting Information [file ADVS-10-2204512-s001.pdf]

ADVANCED SCIENCE
Open Access

Supporting Information

for *Adv. Sci.*, DOI 10.1002/advs.202204512

PbI_2-DMSO Assisted In Situ Growth of Perovskite Wafers for Sensitive Direct X-Ray Detection

Wenjun Liu, Tongyu Shi, Jiongtao Zhu, Zhenyu Zhang, Dong Li, Xingchen He, Xiongsheng Fan, Lingqiang Meng, Jiahong Wang, Rui He, Yongshuai Ge, Yanliang Liu*, Paul K. Chu and Xue-Feng Yu**

Supporting Information

PbI_2-DMSO Assisted *In Situ* Growth of Perovskite Wafers for Sensitive Direct X-ray Detection

Wenjun Liu, Tongyu Shi, Jiongtao Zhu, Zhenyu Zhang, Dong Li, Xingchen He, Xiongsheng Fan, Lingqiang Meng, Jiahong Wang, Rui He, Yongshuai Ge, Yanliang Liu*, Paul K. Chu, Xue-Feng Yu**

W. J. Liu, T. Y. Shi, Z. Y. Zhang, D. Li, X. C. He, L. Q. Meng, X. S. Fan, R. He, Prof. J. H. Wang, Prof. X.-F. Yu, Prof. Y. L. Liu

Materials Interfaces Center, Shenzhen Institute of Advanced Technology, Chinese Academy of Sciences, Shenzhen 518055, Guangdong, China

J. T. Zhu, Prof. Y. S. Ge

Research Center for Medical Artificial Intelligence, Shenzhen Institute of Advanced Technology, Chinese Academy of Sciences, 518055 Shenzhen, China

T. Y. Shi, Prof. Y. S. Ge, Prof. J. H. Wang,Prof. Y. L. Liu, Prof. X.-F. Yu

University of Chinese Academy of Sciences, Beijing 100049, P. R. China

W. J. Liu

Nano Science and Technology Institute, University of Science and Technology of China, Suzhou 215123, China

Prof. P. K. Chu

Department of Physics, Department of Materials Science and Engineering, and Department of Biomedical Engineering, City University of Hong Kong, Tat Chee Avenue, Kowloon, Hong Kong, China

*Corresponding authors: E-mail: yl.liu4@siat.ac.cn (Yanliang Liu); ys.ge@siat.ac.cn (Yongshuai Ge) and xf.yu@siat.ac.cn (Xue-Feng Yu).

Experimental Section

Chemicals. Methylammonium iodide (MAI, >99.99%) was purchased from GreatCell Solar and lead iodide (PbI_2, 98%) was bought from Aladdin. The organic solvents such as dimethyl sulfoxide (DMSO, AR, 99%), acetone (AR, 99%), and N,N-dimethylformamide (DMF, AR, 99%) were supplied by Sigma-Aldrich. [6,6]-phenyl C61 butyric acid methyl (PCBM, AR, 99%) was provided by Xi'an Polymer Light Corporation. The gold particles (product No. Au11474 3*3mm) used for gold electrode preparation were purchased from Zhong Nuo New Materials Co., Ltd. (Beijing, China). All these chemicals were used as received.

Synthesis of PbI_2-DMSO compound. 4.61 g of the yellow PbI_2 powder were dissolved in 10 mL of DMSO to form a PbI_2 solution. The solution was stirred until it became clear. 1 ml of lead iodide dimethyl sulfoxide was added to 50 ml of acetone to form white flocs. The precipitate was obtained by filtration and dried in a vacuum at 40 ℃ to obtain pure white powder.

Synthesis of $MAPbI_3$ powder. The $MAPbI_3$ microcrystals were synthesized by the anti-solvent assisted precipitation method. CH_3NH_3I (0.6358g, 4 mmol) and PbI_2 (1.8440g, 4 mmol) crystals with a ratio of 1:1 were dissolved in 5 ml DMF to form a 4 mM solution. 1 ml of the mixture was injected into 15 ml of chloroform to form $MAPbI_3$ nanocrystalline precipitates within a few seconds. After standing for a period of time, the microspheres were washed twice with chloroform in a centrifuge

and dried in a vacuum oven at 50 ℃ overnight.

Preparation of the $MAPbI_3$ wafer. 0.41 mg of MAI, 1.40 mg of PbI_2-DMSO, and 180 mg of $MAPbI_3$, were weighed and mixed. The mixed powders were then filled to a pie shape (dimensions of 1 cm × 1 cm) and pressed to 10 MPa at 100℃ for 90 min. The heat-assisted pressing process produced the black perovskite wafer with a mirror surface. Thereafter, the wafer was annealed at 100 ℃ for 30 min in a glovebox to evaporate residual DMSO and further crystallized.

Device fabrication. The perovskite X-ray detector had the device structure of Au (80 nm) / PCBM / perovskite wafer / Au (80nm). The PCBM layer was deposited onto the perovskite wafer by spin-coating a PCBM solution (20 mg/mL) at 1,500 rpm for 30 s. The Au films were deposited onto both sides by thermal evaporation. The X-ray detector had an effective device area of 0.0314 cm^2 (1 mm × 1 mm × π) defined by the overlapping area of the top and bottom Au electrodes.

Materials characterization. The morphology of the $MAPbI_3$ powder and wafer were examined by the scanning electron microscopy (SEM, Zeiss GeminiSEM 300) and atomic force microscopy (Bruker, USA). The structure and component of the powder were analyzed by Fourier-transform infrared spectroscopy (FT-IR, Thermo Scientific, Nicolet iS50) using the KBr pellet method. Powder X-ray diffraction (XRD) was performed on the Rigaku Smartlab 3kW X-ray diffractometer with Cu K_α

radiation (λ = 1.54056 Å, 40 kV, 30 mA, 10° min^{-1} from 10 to 60°). The UV–vis absorption spectra were obtained on a UV–vis spectrophotometer (UV-1800, Shimadzu). Raman scattering was carried out on the Horiba Jobin Yvon LabRam HR-VIS high-resolution confocal Raman microscope equipped with a 633 nm laser. The PL and TRPL properties were determined by fluorescent spectrophotometry (Hitachi F-4600 and Edinburgh FLS-1000) using an excitation wavelength of 365 nm. The PL spectra were automatically recorded every 100 ms during the PL measurement. The time-resolved photoluminescence (TRPL) spectra were recorded by a time-correlated single-photon counting spectrometer (Horiba, FluoroMax-4) with an excitation source of 365 nm laser.

Device characterization. The X-ray detection properties of the detector were evaluated using an X-ray generation system for medical imaging (Varex, G242, 18932-M8, USA). The accelerating voltage was 50 kV and the currents were varied from 10 to 200 uA. The dose rate of the X-rays was strictly calibrated using an X2 CT dosimeter (Unfors Raysafe, Sweden). During the measurement, the environment was kept dark and the external electrical bias and current were recorded by the PDA FS380 semiconductor analyzer. The X-ray imaging capability of the detector was demonstrated by moving the investigated object between the detector (3.14 mm^2) and X-ray beam (1.62 mGy_{air} s^{-1}) using a self-assembled x-y scanning system. The home-made x-y scanning system consisted of a motorized linear displacement stage (Newport, M-IMS400CC. A motorized linear displacement stage combined with a

motion controller (Newport, M-IMS400CC) was used to control the scanning along the x and y axes.

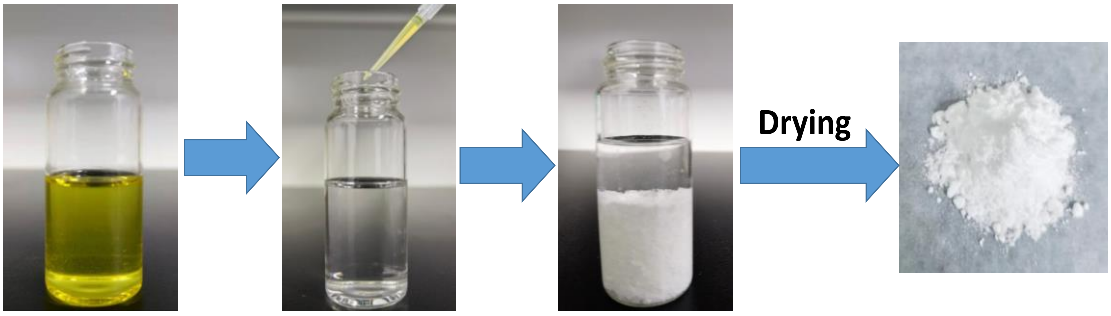

Figure S1. Photographs showing synthesis of the PbI_2-DMSO powder.

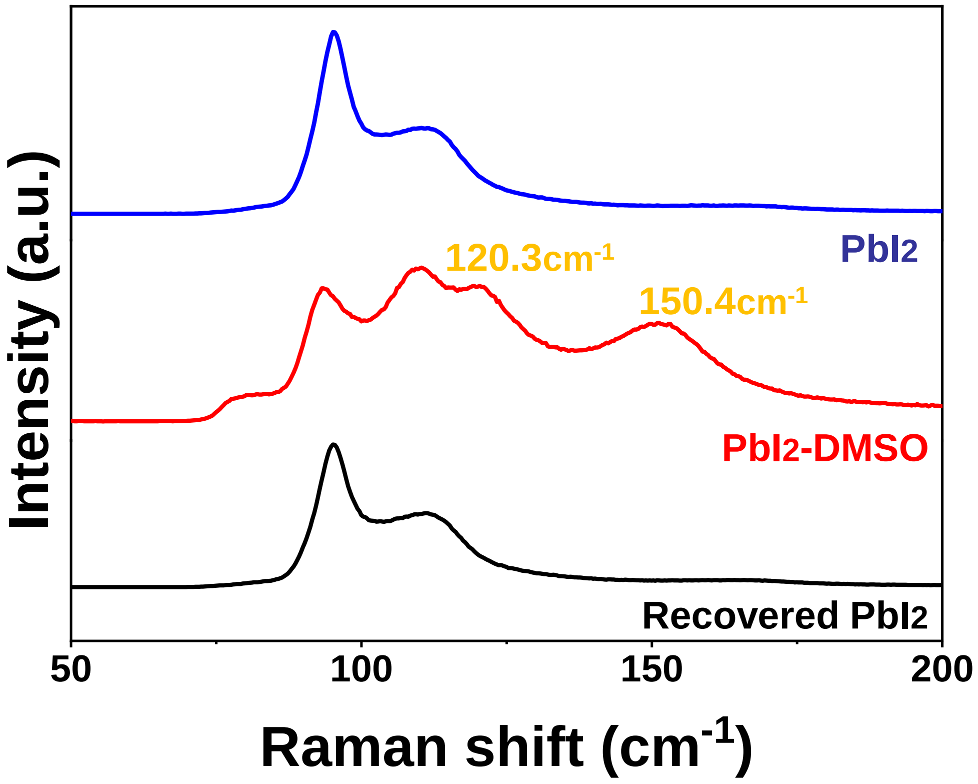

Figure S2. Raman scattering spectra of PbI_2, PbI_2-DMSO, and recovered PbI_2.

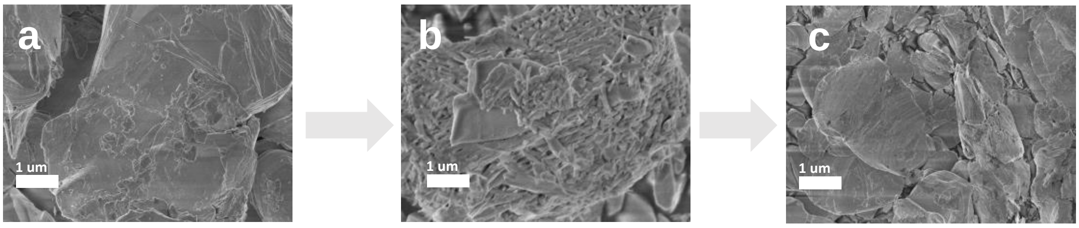

Figure S3. SEM images of (a) Pristine PbI_2, (b) PbI_2-DMSO, and (c) Recovered PbI_2 powders.

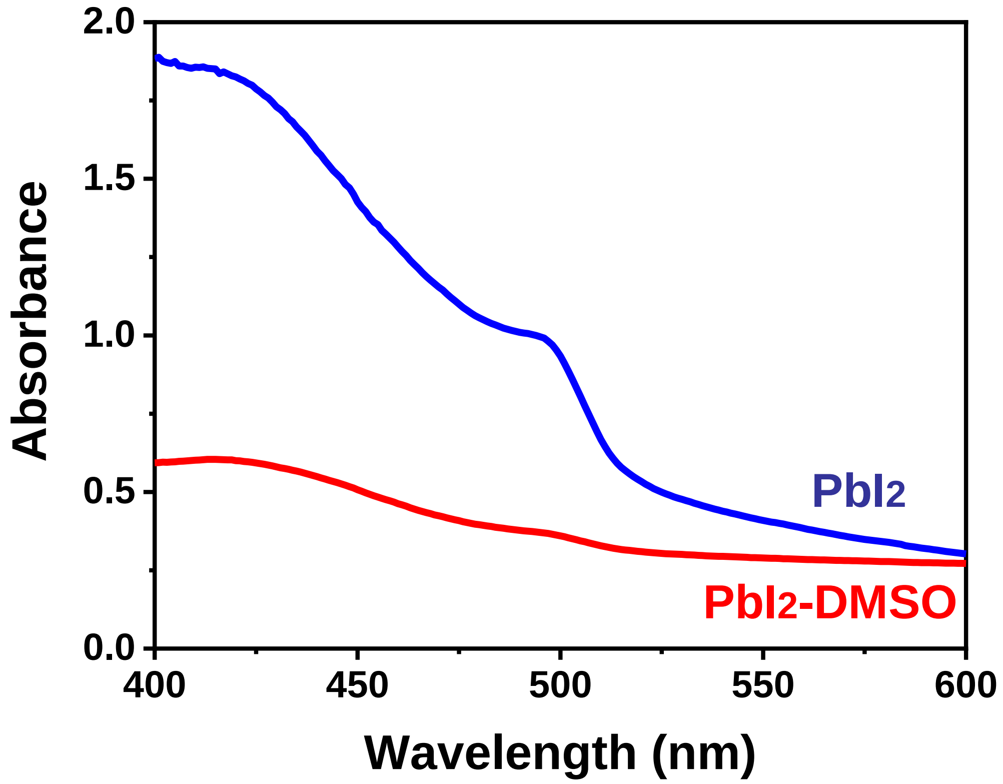

Figure S4. UV-visible spectra of PbI_2 and PbI_2-DMSO.

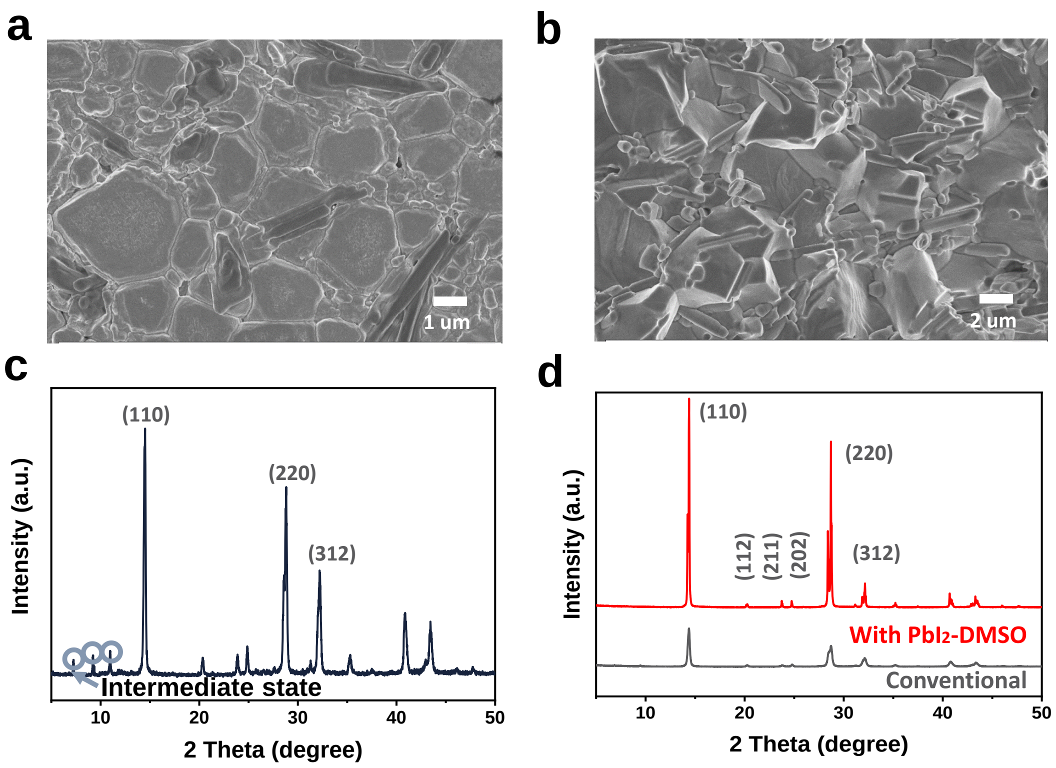

Figure S5. (a) top-view and (b) cross-section SEM images of intermediate $MAPbI_3$ wafer, (c) XRD pattern of intermediate $MAPbI_3$ wafer, (d) XRD patterns of the conventional and PbI_2-DMSO assisted $MAPbI_3$ wafer.

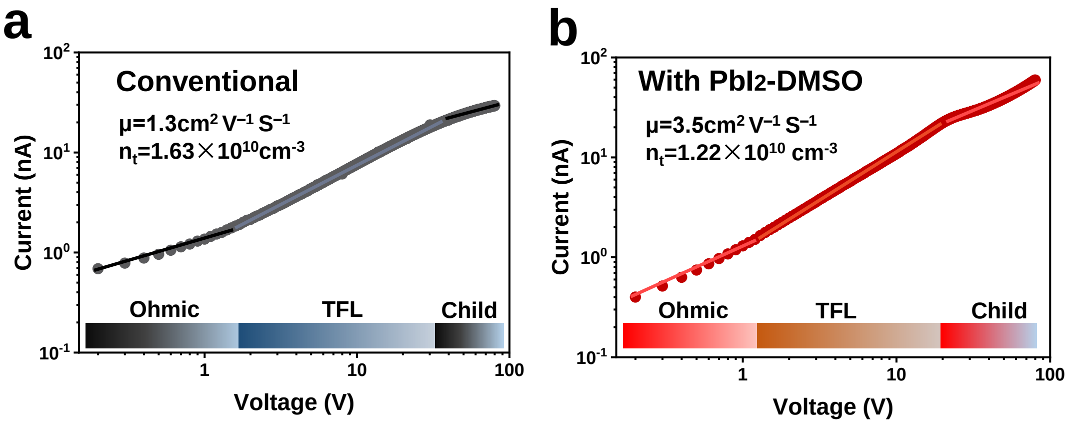

Figure S6. The space charge-limited current (SCLC) measurements of the conventional and PbI_2-DMSO assisted $MAPbI_3$ wafers.

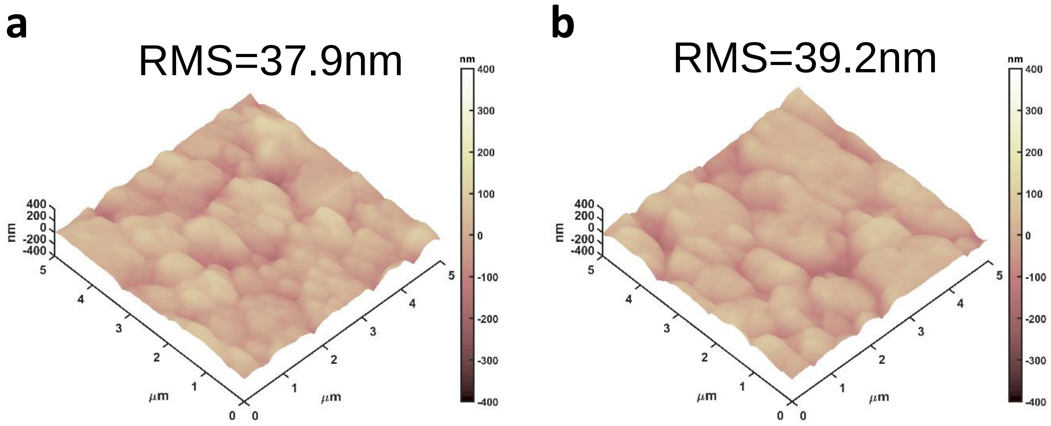

Figure S7. AFM images of (a) Conventional $MAPbI_3$ wafer and (b) 1% PbI_2-DMSO assisted $MAPbI_3$ wafer.

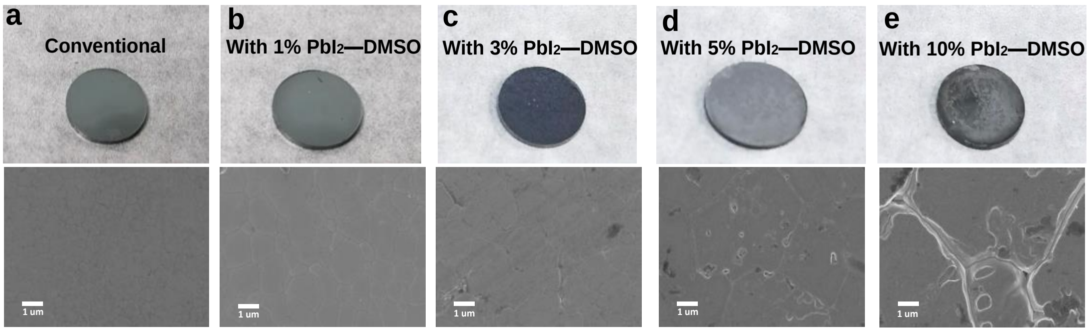

Figure S8. (a-e) Photographs and SEM images of the conventional and PbI_2-DMSO assisted $MAPbI_3$ wafers with various weight ratio.

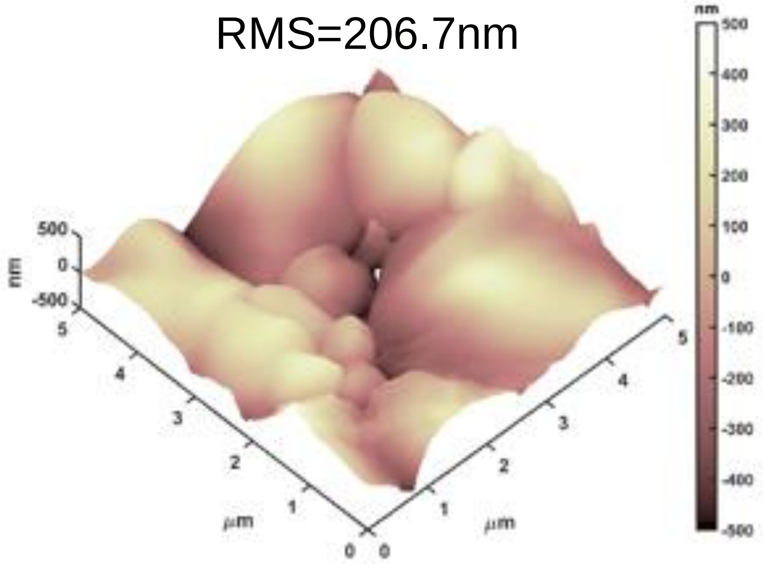

Figure S9.AFM image of the 10% PbI_2-DMSO assisted $MAPbI_3$ wafer with excess additive.

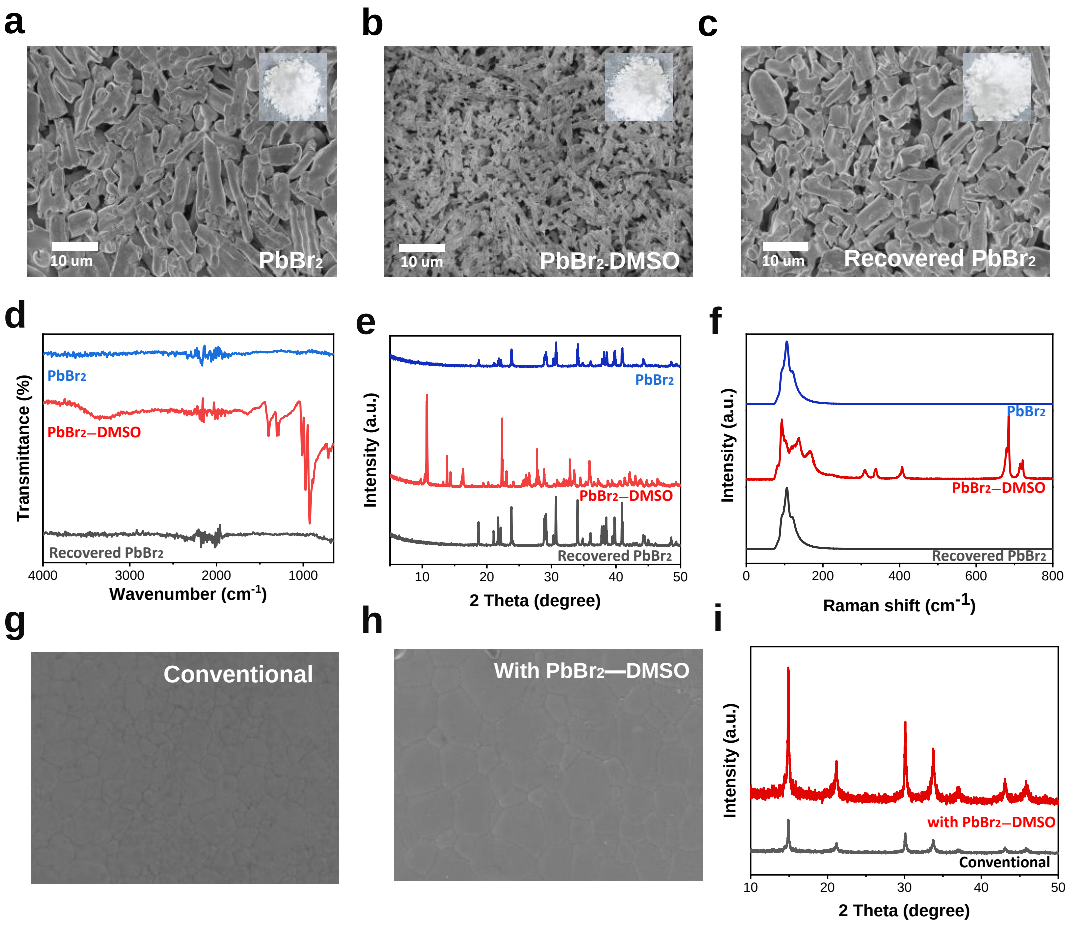

Figure S10. SEM images and photos of (a) original $PbBr_2$ (b) $PbBr_2$-DMSO complex compound and (c) recovered $PbBr_2$ powders, the (d) FTIR (e) XRD and (f) Raman spectra of $PbBr_2$, $PbBr_2$-DMSO and recovered $PbBr_2$-DMSO. The (g, h) SEM images and (i) XRD pattern of the conventional and $PbBr_2$-DMSO assisted $MAPbBr_3$ wafer.

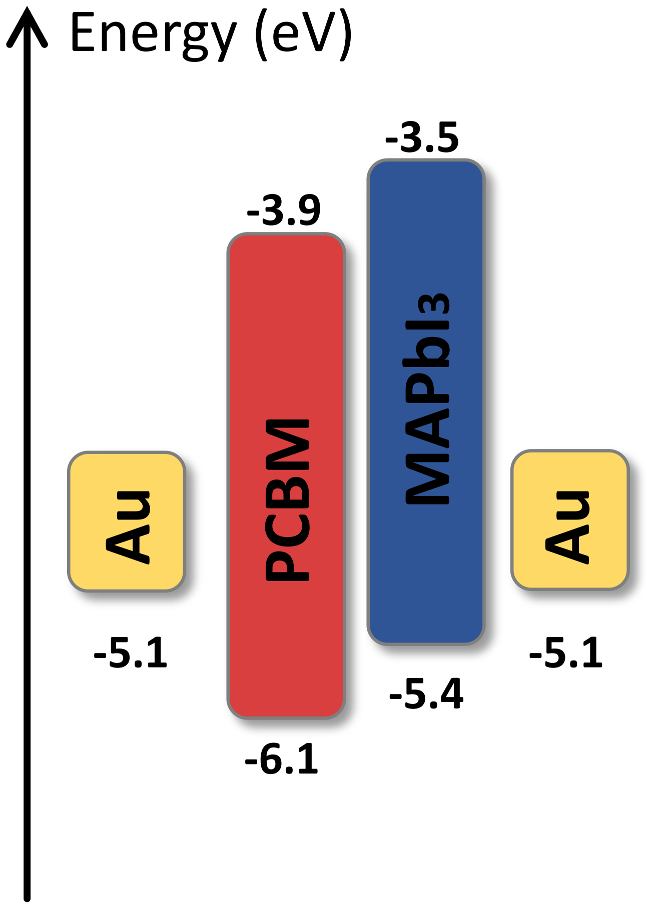

Figure S11. Energy level diagram of the perovskite X-ray detector.

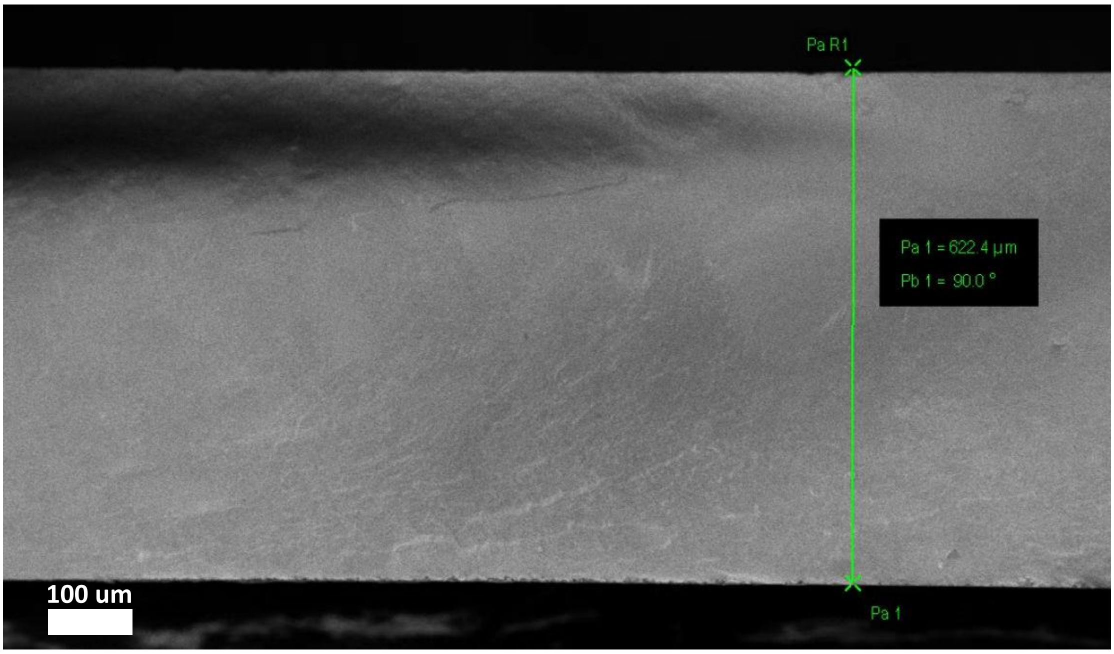

Figure S12. Thickness of the *in situ* grown $MAPbI_3$ wafer.

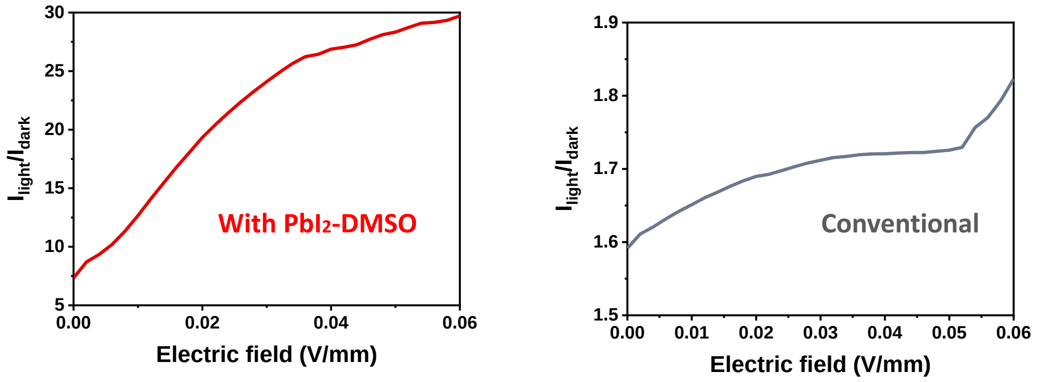

Figure S13. Switching ratios (I $_{light}$ / I $_{Dark}$) of the conventional and PbI_2-DMSO assisted $MAPbI_3$ X-ray detectors.

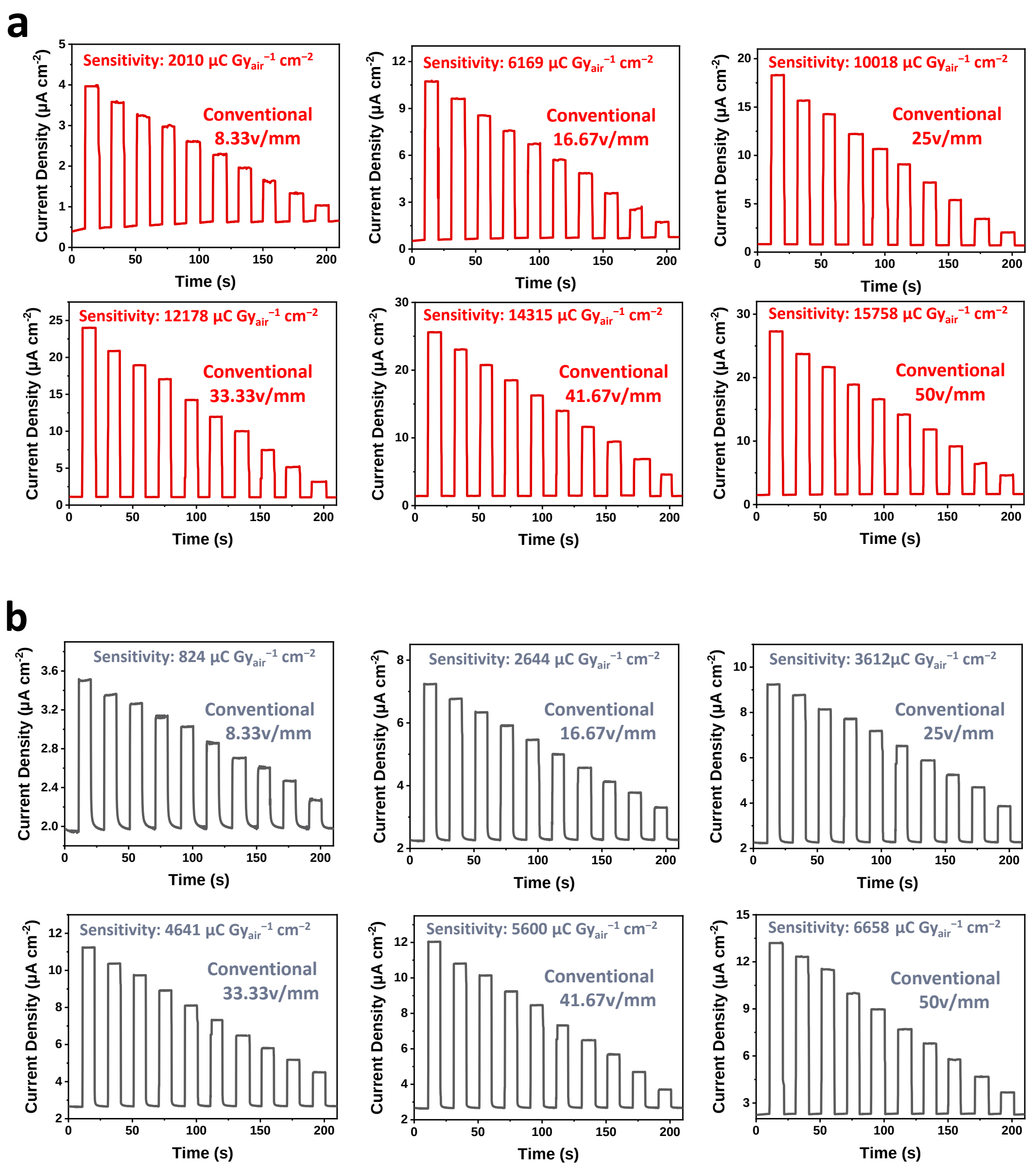

Figure S14. Response to X-ray by turning the X-ray source on and off for different dose rates at biases if 8.33, 16.67, 25, 33.33, 41.67, and 50 V/mm.

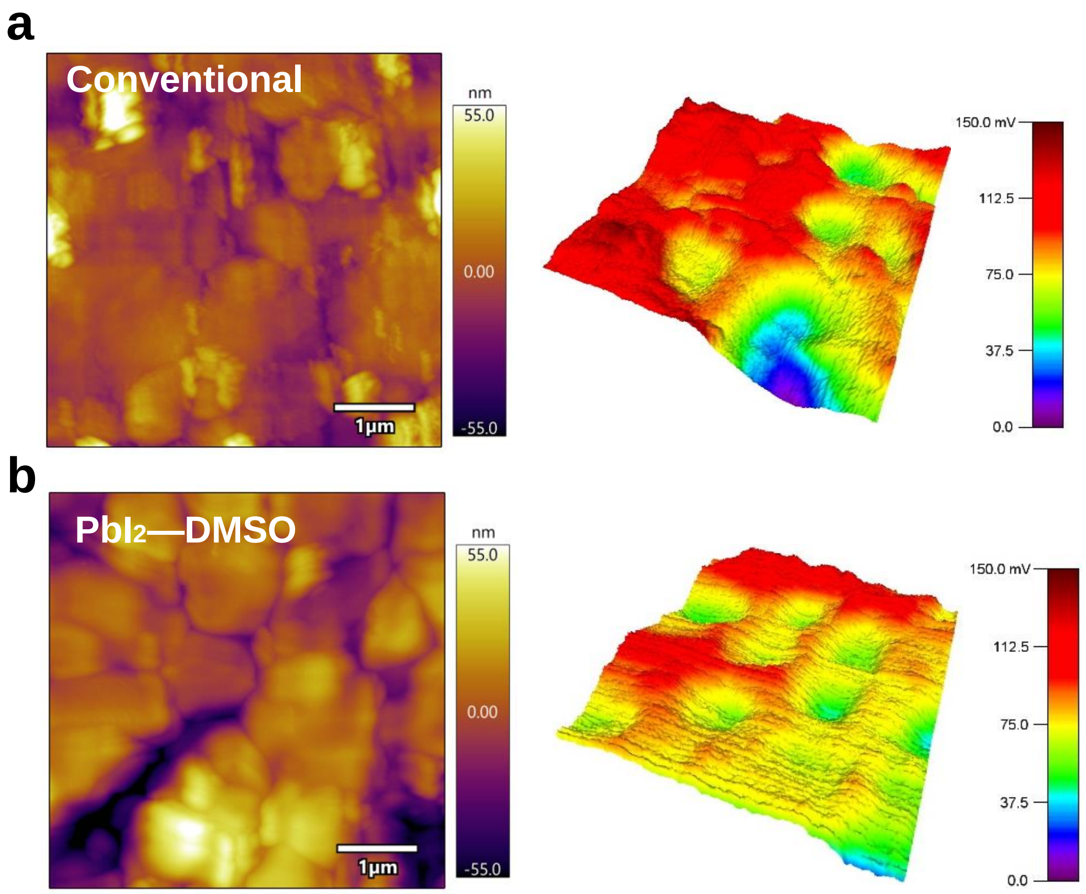

Figure S15. Kelvin probe force microscopy (KPFM) images of conventional and PbI_2-DMSO assisted $MAPbI_3$ wafers.

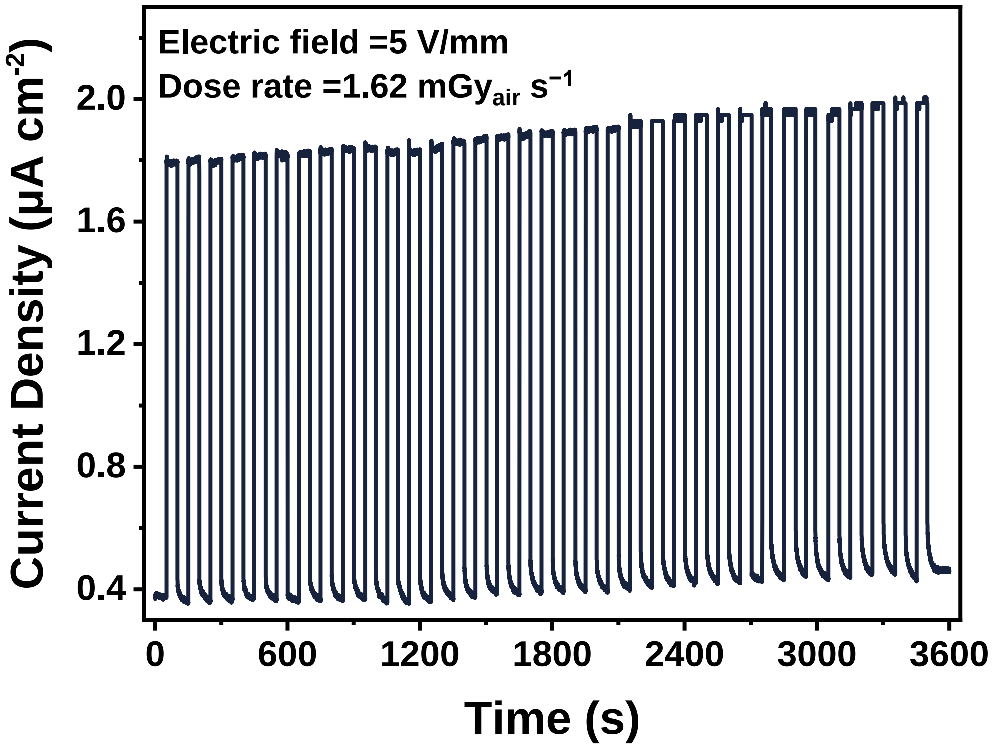

Figure S16. Stability of the PbI_2-DMSO assisted detector under pulsed X-ray irradiation with a fixed dose rate.

Table S1. AFM parameters of the conventional and PbI_2-DMSO assisted $MAPbI_3$ wafers.

**Samples**	**R_q (nm)**	**R_a (nm)**	**Aspect ratios**
Conventional	37.9 ± 15.6	36.1 ± 12.5	1.141 ± 0.068
PbI_2-DMSO assisted (1 %)	39.2 ± 7.8	36.4 ± 7.2	1.074 ± 0.004
PbI_2-DMSO assisted (10 %)	206.7 ± 49.5	171.8 ± 42.3	1.282 ± 0.080

Table S2. PL decay profiles of the conventional and PbI_2-DMSO assisted $MAPbI_3$ wafers fitted with thebi-exponential function, $\boldsymbol{I(t) = A_1 \exp(-t/\tau_1) + A_2 \exp(-t/\tau_2)}$, where $\boldsymbol{I(t)}$ is the time-dependent PL intensity and $\tau_{ave} = (A_1{}^2\tau_1{}^2+A_2{}^2\tau_2{}^2)/(A_1\tau_1+A_2\tau_2)$.

**Samples**	A_1	τ_1 **[ns]**	A_2	τ_2 **[ns]**	T_{avg}
Conventional	0.48	24.31453	0.52	55.40395	23.86873291
PbI_2-DMSO assisted	0.45	50.74884	0.55	68.60182	32.55852048
